# Phytotoxicity and oxidative stress perspective of two selected nanoparticles in *Brassica juncea*

**DOI:** 10.1007/s13205-016-0550-3

**Published:** 2016-11-15

**Authors:** Sunita Rao, Gyan Singh Shekhawat

**Affiliations:** 1 Department of Science, Biyani Girls College, Jaipur, Rajasthan 302023 India; 2 Department of Botany, Jai Narain Vyas University, Jodhpur, Rajasthan 342008 India

**Keywords:** Nanoparticles, Ascorbate peroxidase, Catalase, Superoxide dismutase, XRD

## Abstract

This study elaborates the consequences of oxidative stress caused by copper oxide (CuO) and titanium dioxide (TiO_2_) nanoparticles (NPs) in *Brassica juncea*. Effect of these two NPs on plant physiology, reactive oxygen scavenging enzyme system (ascorbate peroxidase, catalase, superoxide dismutase), proline content and lipid peroxidation has been estimated in leaves as well as root tissues. Bioaccumulation of NPs has also been evaluated in the current study and the interrelated cascade of the enzymatic system with H_2_O_2_ production was identified. The uptake of NPs in plant leaves was confirmed by scanning electron microscopy, X-ray diffraction, and Fourier Transform Infrared Spectroscopy. Plant growth was found to be diminished with elevated levels of CuO NPs whereas TiO_2_ NPs had shown an opposite effect. The plant species accumulated lower concentration of NPs and displayed considerable tolerance against stress, probably due to well-organized and coordinated defense system at the root and shoot level by the intonation of antioxidative enzymes.

**Electronic supplementary material:**

The online version of this article (doi:10.1007/s13205-016-0550-3) contains supplementary material, which is available to authorized users.

## Introduction

Nanoparticles have received a tremendous attention for their optimistic impact in many sectors of research and development. In particular, CuO NPs are being used and marketed as antifouling paints for boats, commonly utilized by ink, plastic, ceramic, electronic and chemical industries (Cioffi et al. 2005; Saison et al. 2010; Pan et al. 2010). Similarly, TiO_2_ NPs are used in medicinal formulations due to germicidal and antimicrobial properties. It is also well-known for making corrosion-proof surfaces of metals (Wold 1993; Ellsworth et al. 2000). The ongoing production and application of metal oxide NPs have increased, due to which possibility of exposure to plants via aerial or root path has been elevated. Once adsorbed to plant surfaces, exact behavior of the NPs inside plant system is still not well-explored because different NPs can lead to either positive or negative effects on the plant system. Toxic response of NPs has been induced by changes in the plant metabolism (Limbach et al. 2007). Retardation in growth and other harmful effects on plants could be correlated with the generation of reactive oxygen species (ROS) in the plant system which results into oxidative stress. Plants may respond to oxidative stress by enzymatic ROS scavenging systems including ascorbate peroxidase (APX), catalase (CAT), and superoxide dismutase (SOD). Among ROS compounds, H_2_O_2_ is the central hub of various reactions within a plant system and has a relatively long half-life (1 ms) and its small size allows traversing through cellular membranes and migration in different compartments, which facilitates its signaling functions (Bienert et al. 2006).

In future more studies focusing on bioaccumulation, biomagnifications and biotransformation of NPs in plant species are required (Rico et al. 2011). Till date, scanty information is available on the interaction of plant species with respect to the accumulation and subsequent availability of NPs in food crops. For the terrestrial plants, uptake of CuO NPs by wheat and toxicity of these NPs to ryegrass, radish has been reported (Zhou et al. 2006; Atha et al. 2012). Another report has shown that TiO_2_ NPs could have a positive effect on the growth of spinach (Tripathi et al. 2016). Likewise, a mixture of nanoscale SiO_2_ and TiO_2_ could speed up the germination and growth in soybean (Lu et al. 2002). Phytotoxicity of NPs on plants was reported in cabbage, carrot, radish, rape, and rye grass (Yang and Watts 2005; Lin and Xing 2007). On the other hand, application of TiO_2_ (size 25 and 100 nm) to willow plant cuttings did not show any significant toxic effects. Furthermore, it was observed that amount of aggregate formation and sedimentation was higher with the increasing particle sizes (Seeger et al. 2009).

To the best of our knowledge, so far no study has been done to investigate the role of antioxidant enzymes against NPs. This study is a dedicated attempt in this direction to investigate the oxidative stress in *Brassica juncea* induced by two different metal oxide nanoparticles. For this purpose a hydroponic system has been used to examine the accumulation and uptake of NPs by *B. juncea*. Additional, study dealing with generation of oxidative stress due to CuO and TiO_2_ NPs in comparison to H_2_O_2_ content has been performed and an interrelated cascade of the enzymatic system with H_2_O_2_ production was identified. Evaluation of the toxic effect of these nanomaterials will not only help to ensure the safety of their wide range of applications, but will also be useful in designing a functional materials with minimal adverse effects to plants and ecosystem.

## Materials and methods

### Sources of CuO and TiO_2_ nanoparticles

Nano-scale copper (II) oxide (CuO) and Titanium dioxide (anatase) TiO_2_ NPs were purchased from Sigma–Aldrich Co. (USA) as shown in Table 1. Appropriate amount of NPs were suspended in Hoagland’s nutrient solution and sonicated to prepare stock solutions (Hoagland and Arnon 1950).

**Table 1:** Characteristic of metal oxide nanoparticles

Product name	Copper (II) oxide, CuO	Titanium (IV) oxide, TiO_2_
CAS-No.	1317-38-0	1317-70-0
EC No.	215-269-1	215-280-1
Color	Black	White
Rel. density	6320 g/cm^3^	39 g/ml
Purity (%)	99.7	99.7
Surface area (m^2^/g)	29	45–55
Size (nm)	<50	<25

### Nanoparticle characterization

To determine the size of CuO and TiO_2_ NPs, X-ray diffraction (Siemens XRD D5000) at room temperature, with CuKα radiation by accelerating voltage of 40 kV at current 40 mA was performed. The average crystalline size of the samples was calculated according to Debye–Scherrer formula *D* = 0.9 *λ*/*β* cos*θ*. Where, *λ* is the wavelength of X-ray radiation, *β* is the full width at half maximum (FWHM) of the peaks at the diffracting angle *θ* (Patterson 1939). The samples were later on analyzed under transmission electron microscopy (TEM) and scanning electron microscopy (SEM) analysis after dispersing in the deionized water by ultrasonic vibration (Sonoplus Ultrasonic Homogenizer HD 2200, Germany). Which was observed by TEM placing a drop onto a carbon-coated copper grid (model: JEOL JEM 1011) operating at 80 kV, using a CCD camera (Hitachi H-7600, AMT V600). Whereas for SEM, gold coating (ca. 1 nm thickness) of samples was done using a sputter coater (Cressington model 108, Ted Pella Inc.), observed with a SEM (model: JEOL 6320FXV).

### Plant growth conditions

*Brassica juncea* (Bio-902) seeds were sterilized by 5% (v/v) sodium hypochlorite solution. Germination of seeds was carried out in glass Petri dishes (10 cm) at 30 °C in dark. Germinated seeds were transferred to thermostatically controlled culture room maintained at 25 ± 2 °C and at 50% relative humidity. The deionized water was replaced with Hoagland’s nutrient solution and seedlings were provided with photosynthetic photon flux density (PPFD) at 500 µM m^−2^ s^−1^ for 14 h daily.

### Nanoparticle treatment on plants

After 13 days of growth, Hoagland solution was replaced with deionized water again and the system was flushed for 2 days. A standard Hoagland’s preparation of CuO and TiO_2_ nanoparticle solution was circulated through the system. On the basis of a preliminary screening of effect on the shoot length, root length; the fresh and dry weight of *B. juncea* seedlings (data not shown). TiO_2_ and CuO NPs solution was provided (i.e., 0–1600 mg/L) to plant and 4 sub-lethal concentrations were selected along with a control. Plant growth conditions were maintained as described in section “Plant growth conditions”.

### Analysis of nanoparticle interaction with plant system

Leaves of plants were washed in deionized water for 3–4 times and dried at 80 °C for 72 h. A powdered sample was prepared and set uniformly on a glass slide for XRD studies. The Fourier Transform infrared spectroscopy (Bruker Alpha- ECOATR) along with attenuated total reflectance (ATR) was used to determine physicochemical and intermolecular cross-linking of NPs and plant system using dried plant root sample homogenized, and placed over the ZnSe crystal of ATR (Hashim et al. 2010).

The anatomy of plant leaf and root were observed using different techniques, such as light microscopy and SEM. Samples collected, were first fixed in 2.5% glutaraldehyde in 0.05 M potassium phosphate buffer (pH-7.1) for 8 h, and dehydrated in an increasing order ethanol series (Sass 2007; Johansen 1940). Root samples were first observed under light microscope (OLYMPUS, CH20*i*) for preliminary observation. The samples were sealed in parafilm, frozen in liquid nitrogen and fractured transversely using a pre-cooled knife, and later observed by SEM.

### Determination of physiological Indexes and biochemical parameters

The concentration of CuO and TiO_2_ NPs in the leaf and root tissues of treated seedlings was measured by atomic absorption spectrophotometer, as described by Brooks and Naidu (1985). Proline content was estimated by the method (Bates et al. 1973). The concentration of proline was determined using the spectrophotometric standard curve of l-Proline (0–100 μg ml^−1^). Lipid peroxidation was estimated by measuring the formation of malondialdehyde (MDA) content with 2-thiobarbituric acid (TBA) according to De Vos et al. (1989). Plant shoot and roots (300 mg) were homogenized in 10 ml of 0.25% (2-thiobarbituric acid) made in 10% trichloroacetic acid (TCA). The level of lipid peroxidation was expressed using extinction coefficient (*ε*) 155 mM^−1^ cm^−1.^

### Determination of H_2_O_2_ content

H_2_O_2_ contents of both control and treated plants were determined according to Loreto and Velikova (2001). Shoot and root samples (100 mg each) were homogenized at 4 °C in 2 ml of 0.1% (w/v) trichloroacetic acid (TCA). The H_2_O_2_ content of the supernatant was evaluated by comparing its absorbance at 390 nm with a standard calibration curve. The H_2_O_2_ content was expressed as µmol g^−1^ fresh weight.

### Enzyme extraction and estimation of antioxidant enzyme activity

Plant shoot and roots samples (500 mg) were homogenized in 50 mM phosphate buffer (pH 7.0) containing 1 mM EDTA, 0.05% Triton X-100, 1 mM polyvinylpyrrolidone and 1 mM ascorbate. After centrifugation at 5,000*g* for 20 min at 4 °C, the supernatant was used for the estimation of antioxidant enzymes. The protein content in the homogenate was estimated by the method of Lowry et al. (1951).

APX (EC 1.11.1.11) activity was determined in a reaction mixture containing 50 mM phosphate buffer (pH 7.0), 0.6 mM ascorbic acid and enzyme extract (Chen and Asada 1989). The addition of 10 μl of 10% H_2_O_2_ to the reaction mixture was done. The decrease in absorbance due to the reaction was recorded at 290 nm for 3 min (ε-2.8 mM^−1^ cm^−1^).

CAT (EC 1.11.1.6) activity was assayed in the presence of H_2_O_2_ by monitoring the decrease in absorbance at 240 nm, as H_2_O_2_ was utilized as substrate. Enzyme activity was evaluated using the extinction coefficient of 0.04 μM^−1^ cm^−1^ for H_2_O_2_ (Aebi 1974).

SOD (EC 1.15.1.1) activity was measured spectrophotometrically at 560 nm as per Beuchamp and Fridovich (1971). Absorbance was recorded at 560 nm (ε-100 mM^−1^ cm^−1^).

### DAB dye staining

For visual detection of the presence of H_2_O_2_ in the leaves 3, 3-diaminobenzidine (DAB) was used as substrate (Orozco-Cárdenas and Ryan 1999). Leaves were at first rinsed with distilled water and were immersed in DAB (1 mg ml^−1^) dye for 24 h at 27 °C. To verify the specificity of spots, before staining with DAB some leaves were incubated for 2 h in 1 mM ascorbate, a H_2_O_2_ scavenger. The H_2_O_2_ staining was repeated three times with similar results. The H_2_O_2_ content was also measured colorimetrically as described by Jana and Choudhuri (1982).

### Data analysis

Each treatment was performed in triplicate, and the results were expressed as mean ± SD (standard deviation). Statistical differences of experimental data were examined by the Student’s *t* test. Each of experimental values was compared with its corresponding control. All the statistical analysis was implemented using SPSS 16.0 (SPSS Inc., Chicago, USA). A significant difference was defined as that with (*p* < 0.001) in all statistical analyses.

## Results and discussions

*Brassica juncea* seedlings were treated with CuO and TiO_2_ NPs at different sub-lethal concentrations (0, 200, 500, 1000, and 1500 mg/L) under controlled conditions in hydroponics for 96 h. After that, seedlings were harvested to study various physiological and biochemical parameters.

### Characterization of NPs

**Fig. 1:**
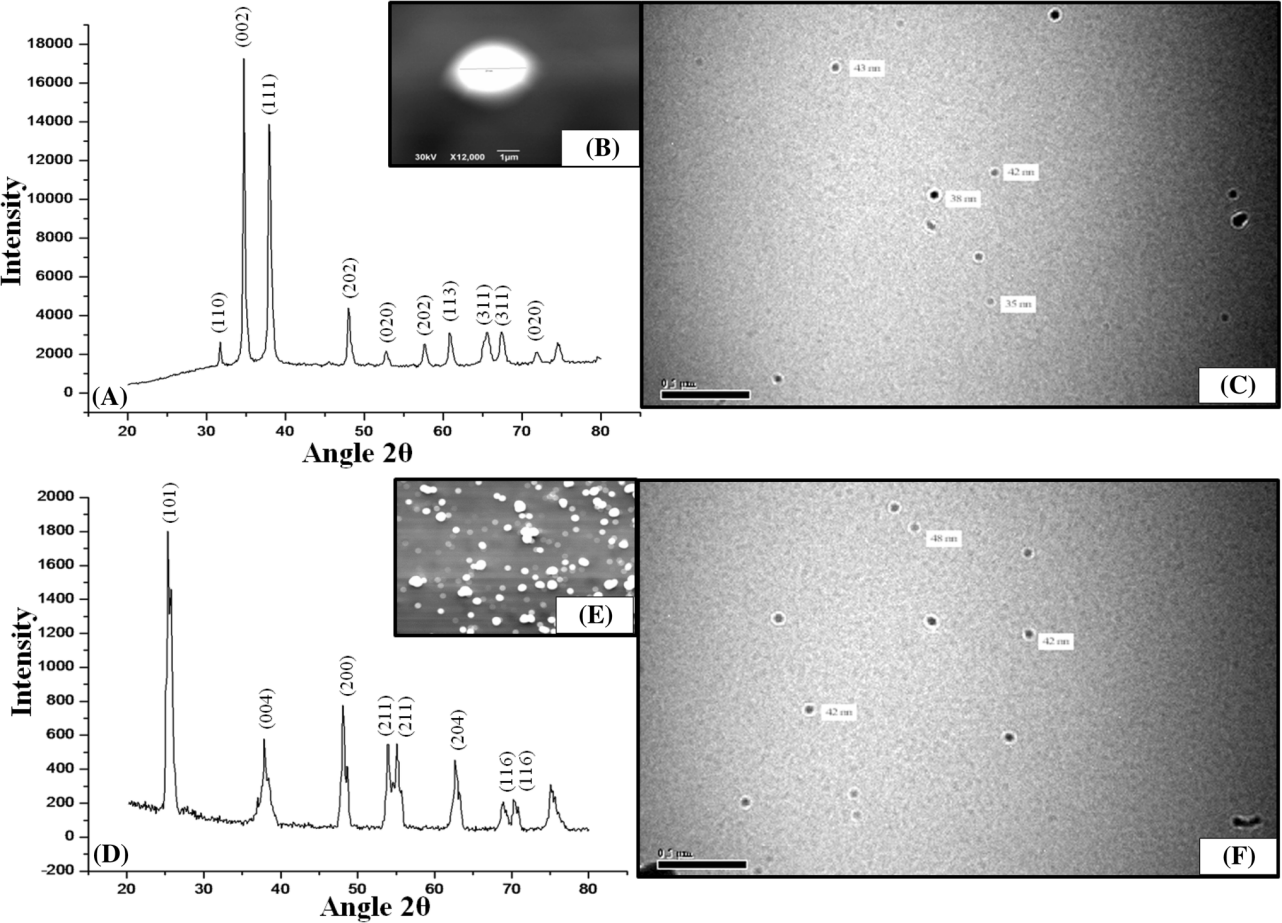
**a** X-ray diffraction pattern of CuO NPs. **b** Morphological characterization of Copper (II) oxide nanoparticles using Scanning electron microscopy (X12,000) at a scale of 1 µM. **c** TEM micrograph of CuO NPs with an average diameter (33 ± 5) nm. **d** X-ray diffractogram of TiO_2_ NPs. **e** SEM image of TiO_2_ NPs showing the spherical morphology. **f** TEM analysis of TiO_2_ NPs with an average diameter (44 ± 4) nm

Characterization of NPs used in bioassays is an essential step because the ability of penetration into plant tissues strongly depends on the physicochemical properties of NPs. Characteristic parts of XRD patterns of CuO and TiO_2_ NPs are summarized in Fig. 1a and d, respectively. All diffraction peaks corresponding to TiO_2_ were well consistent with standard JCPDS anatase patterns. The XRD spectrum of CuO NPs was also confirmed from standard JCPDS database. The morphology of NPs was analyzed by SEM. The obtained SEM image revealed the size and shape of NPs (Fig. 1b, e). TEM image of the CuO NPs revealed the diameter of NPs with a spherical, truncated, and uneven nature with an average size of approximately 39 ± 3 nm as shown in Fig. 1c. Similarly, TiO_2_ too had a spherical shape observed by TEM with a size 44 ± 4 nm as in Fig. 1f.

### Determination of growth parameters

**Fig. 2:**
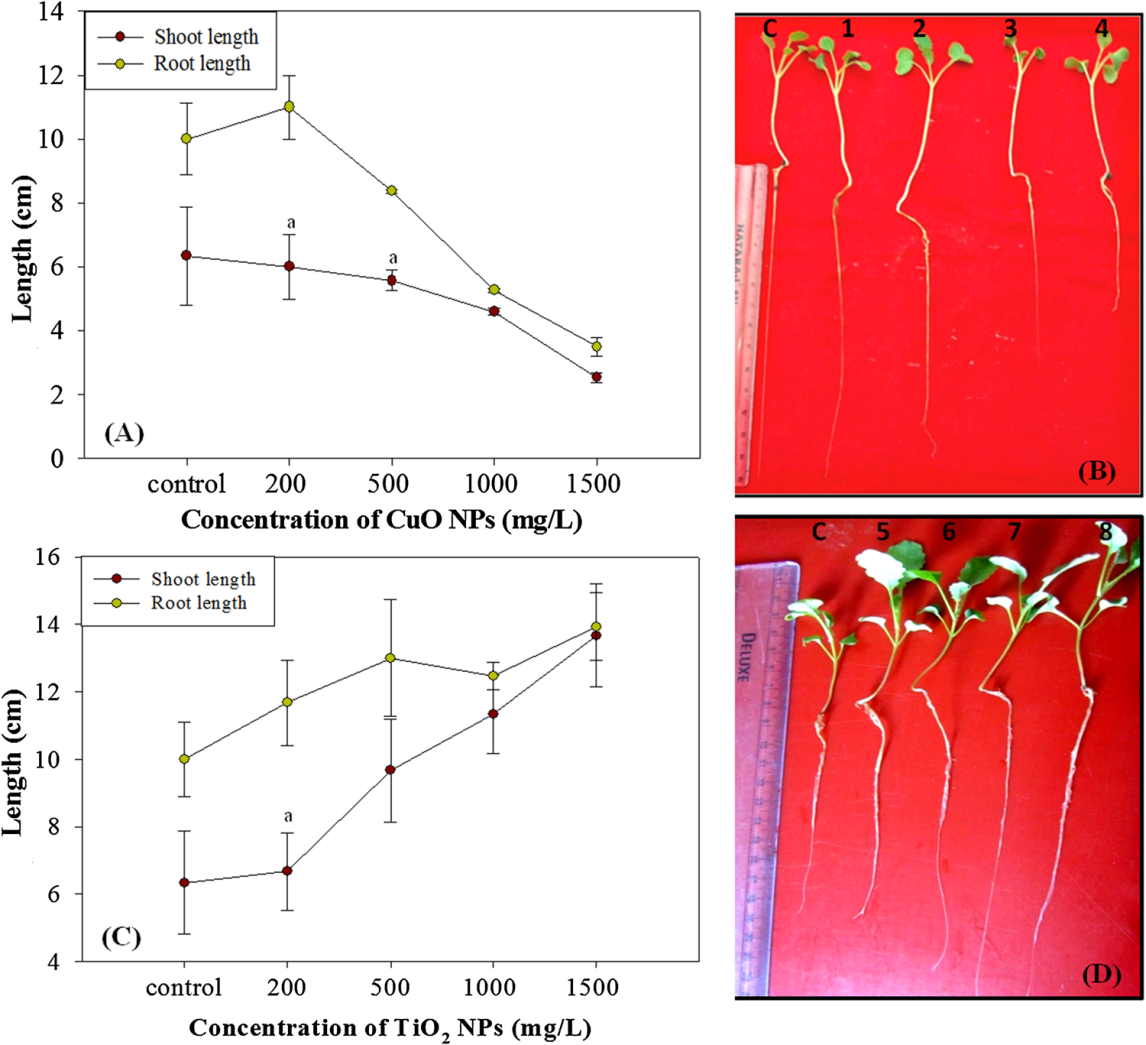
**a** Graphical representation of effect of CuO NPs on shoot and root length of *B. juncea*. **b** Effect of CuO NPs (C, 1, 2, 3, 4) symbolize the plants at different concentrations, i.e., control, 200 500, 1000, 1500 mg/L resp. **c** Effect of TiO_2_ NPs on shoot and root length of *B. juncea.* **d** Effect of TiO_2_ NPs (C, 5, 6, 7, 8) correspond to the plants at different concentrations, i.e., control, 200 500, 1000, 1500 mg/L resp. *Bars* indicate means ± SDs (*n* = 3), and *different letters on bars* indicate no significant differences between treated and control plant set in the respective growth parameters (*p* < 0.001)

To observe the potential effect of CuO and TiO_2_ NPs on the growth of *B. juncea,* 15-day old seedlings were exposed to both of the NPs under the controlled conditions in hydroponics. There was a significant decline in root and shoot length of the plant with elevated NPs concentrations as shown in Fig. 2a. A significant reduction in the growth of seedlings was recorded from 200 to 1500 mg/L CuO nanoparticle treatment (Fig. 2b). Roots of the CuO NPs treated plants were thinner and more brittle than control plants with brown necrotic lesions. The negative effect of CuO NPs on plant growth was noticed as there was a correlation between increasing CuO concentrations with reduced shoot and root length. As plant roots came to direct contact with NPs there could be an aggregation of particles on the root surfaces which might have hindered the nutrient flow, thus inhibiting root growth at higher NP concentrations. Results obtained here were similar to the work by Stampoulis et al. 2009, where five NPs including CuO NPs were studied, which reduced the emerging root length in *Cucurbita pepo* at the concentration 1000 mg/L.

Another study conducted by Lee et al. (2008), demonstrated that CuO NPs were toxic to mung bean and wheat as denoted by reduced seedling growth. Similarly, for 14-day old seedlings of wheat treated with high CuO nanoparticle, 59 and 13% reduction in growth was noticed for root and shoot, respectively, (Dimkpa et al. 2012) indicating dose-dependent phenomenon. In another study, a 50% inhibition of growth of duckweeds was observed upon treatment with CuO NPs (Shi et al. 2011).

On contrary, in this study there was a significant increase in root length except at 1000 mg/L (TiO_2_ NPs) while, the shoot length was found to be significantly better at 500, 1000, 1500 mg/L TiO_2_ NPs (Fig. 2c). These plants had a higher biomass than control plant species due to higher shoot and root length of treated plants (Fig. 2d). Similarly, root length of *Lemna minor* found to be increased with TiO_2_ NPs concentrations lower than 500 mg/L (Song et al. 2012). In wheat, root elongation was significantly 68% higher and in rapeseed root length increased by 31% (Larue et al. 2012). Therefore, it is apparent from above results that same plant system could act differentially against two different NPs of almost equal size even when the concentrations of nanoparticles were same.

### Quantification of CuO and TiO_2_ in dry plant tissues

It was observed that content of NPs in plant tissues was directly proportional to the concentration of NPs in the growth medium. The bioavailability of NPs to the test plants was estimated by calculating the bioaccumulation factor, defined as the NPs concentration in the plants (mg/g DW) divided by the NPs concentration in the growth media (mg/L) as shown in Table 2. TiO_2_ NPs treatment resulted in highest accumulation at a concentration 1500 mg/L which was 90% higher than the control with a bioaccumulation factor 0.022 L g^−1^. Whereas, in CuO treated plants there was a significant increase in content in accordance with the increased NPs in the medium but it was 81% more than the control at 1500 mg/L and bioaccumulation factor was 7.87 L g^−1^. A recent study showed significant uptake of nano-sized copper by mung bean and wheat, with reported bioaccumulation factors of 8 and 32 L kg^−1^, respectively (Lee et al. 2012). Greater accumulation of NPs in the roots of *B. juncea* could be simply explained by its root morphology. The plant has a greater surface area because of its thin and numerous roots facilitating the penetration of more NPs and accumulation which might depend upon the chemical properties of the NPs too. However, it is not surprising, that nanoparticles can explicate their actions depending on the size or shape, applied concentrations, specific conditions of experiments, plant species, and the mechanism of uptake (Castiglione et al. 2011). We propose that although all these factors were kept identical but still properties of the nanoparticles are different which might be responsible for the differential action of both NPs.

**Table 2:** Effect of TiO_2_ and CuO NPs on nanoparticle content, bioaccumulation factor, proline content, malondialdehyde content

Treatment	NP content (mg g^−1^ DW)	Bioaccumulation factor (L g^−1^)	Proline content (µM g^−1^ FW)		MDA content (µM g^−1^ FW)	
TiO_2_	(Shoot + root)	(Shoot + root)	Shoot	Root	Shoot	Root
Control	41.84 ± 2.23	0.00 ± 0.00	30.70 ± 4.2	39.06 ± 5.8	44.14 ± 5.3	18.7 ± 3.2
200 mg/L	153.6 ± 5.07	0.025 ± 0.00	69.89 ± 1.4	56.93 ± 0.8	25.58 ± 1.7	18.1 ± 2.3*
500 mg/L	199.7 ± 2.44	0.018 ± 0.00	72.22 ± 0.8	67.73 ± 3.1	18.20 ± 0.7	12.9 ± 0.7
1000 mg/L	419.6 ± 8.82	0.017 ± 0.00	89.10 ± 0.8	76.96 ± 4.7	17.7 ± 0.8	16.2 ± 0.2*
1500 mg/L	422.9 ± 6.57	0.022 ± 0.00	92.07 ± 6.9	99.73 ± 1.2	22.4 ± 1.2	20.5 ± 0.5
CuO						
Control	34.84 ± 2.2	0.00 ± 0.00	31.80 ± 1.1	38.28 ± 1.1	44.14 ± 5.3	18.7 ± 3.2
200 mg/L	96.08 ± 5.2	2.05 ± 0.09	35.92 ± 3.5	32.78 ± 0.8	18.9 ± 0.5	16.9 ± 1.2*
500 mg/L	94.69 ± 0.6	5.28 ± 0.03	41.42 ± 2.4	44.75 ± 1.1	18.8 ± 0.3	28.0 ± 0.6
1000 mg/L	117.7 ± 1.7	8.49 ± 0.1	69.10 ± 1.7	81.07 ± 0.8	28.5 ± 2.2	96.2 ± 2.5
1500 mg/L	190.4 ± 3.0	7.87 ± 0.1	90.69 ± 4.2	109.9 ± 1.4	22.3 ± 0.9	141.6 ± 2.2

* Means with “asterisk” are not significantly different from control at (*p* < 0.01), according to student’s *t* test

### X-ray diffraction and FTIR analysis

**Fig. 3:**
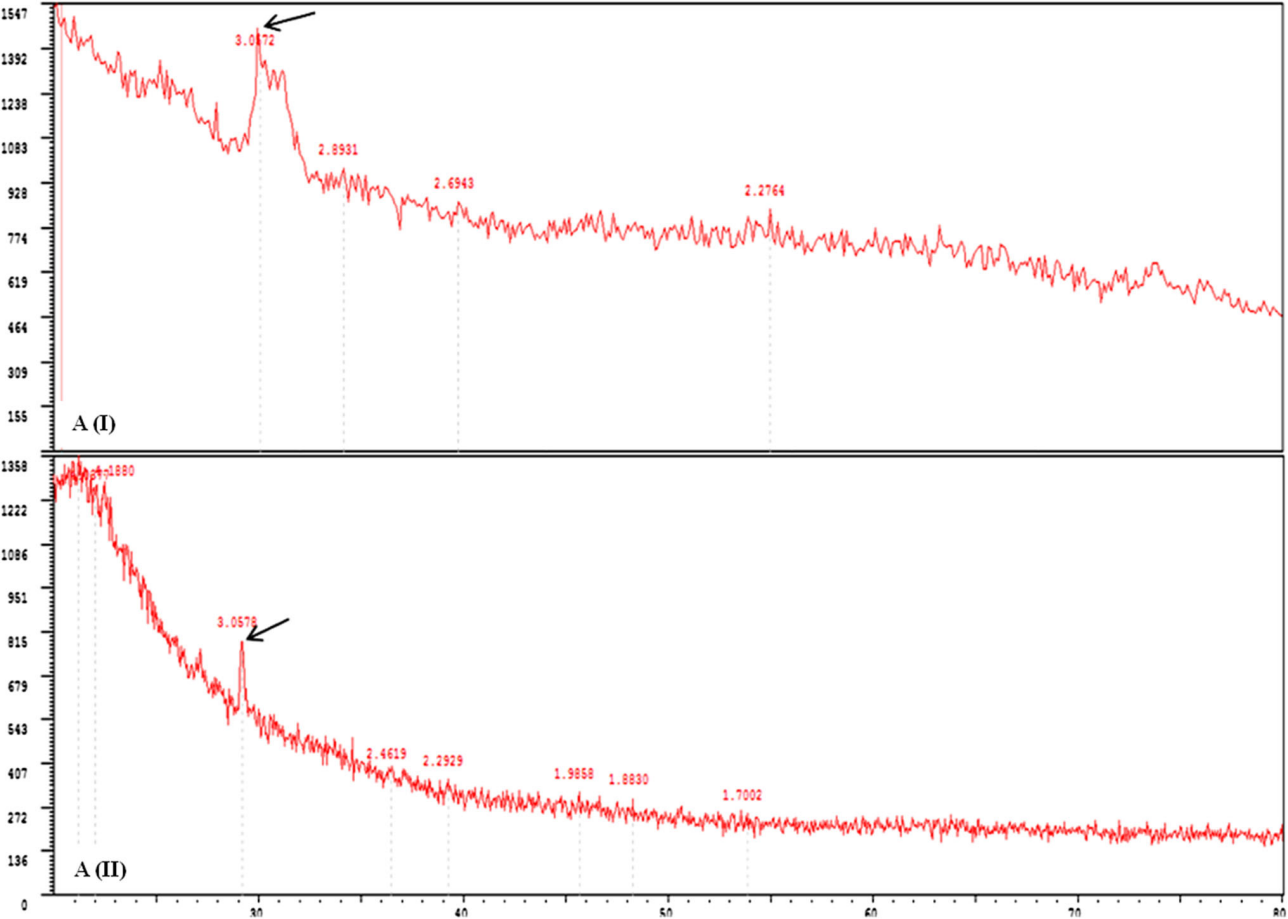
**a(I)** X-ray diffraction pattern of plant root samples exposed to CuO NPs. **a(II)** X-ray diffraction pattern of plant root TiO_2_ NPs. *Marked arrows* indicating the peaks *d*-*spacing* values in the root tissues

XRD analysis of treated leaf (1500 mg/L) at the end of phytotoxicity periods was selected for study. The *d spacing* of the CuO NPs in the leaf of treated plants as shown in Fig. 3a(I) indicated the particles with an average diameter of 2–4 nm. The size of TiO_2_ NPs from Fig. 3a(II) was observed to be of similar to those of CuO (around 2 nm) those were capable of traveling from roots to shoot and up to the leaf.

All molecules have the natural ability to interact with light of different wavelengths. FTIR analysis was performed to find out the possible biomolecular interactions of root samples with nanoparticles. The FTIR spectra of CuO and TiO_2_ (1500 mg/L) NPs obtained a transmittance peaks located in the regions 400–3900 cm^−1^ (Supplement Figure S1). Control plant samples had peaks (red color) at 3280.94, 2096.96 and 1637.14. The FTIR spectra of control compared with the peaks of CuO NPs (pink color) appeared at 1541.19, 2116.55 and few in a range of 3277.70–3852.28 (Supplement Figure S1). The peak at 1410–1467 cm^−1^ was associated with the deformation of aliphatic C–H (Wen et al. 2008). The peak at 1600–1650 cm^−1^ was ascribed to C=O stretching, aromatic C=C, hydrogen bonded C=O, a double bond conjugated with carbonyl and COO^−^ vibrations, and antisymmetrical stretching of COO^−^ groups (Peuravuori et al. 2005; Pavia et al. 2001). The peaks at 3347 were credited to bond O–H. While TiO_2_ treated samples (blue color) spectrum was in the range of, i.e., 1396.29–1558.31, 1635–2108.79 and 3275.31–3901.64 cm^−1^. It was observed in the present study that alteration in the peak at 1630 cm^−1^ (carboxylic like) resulted from NP binding. The emergence of new peaks in treated plant samples as compared to control could be due to the interaction of nanoparticles with organic molecules in the plant.

### Influence of nanoparticles on structural anatomy of *B. juncea*

**Fig. 4:**
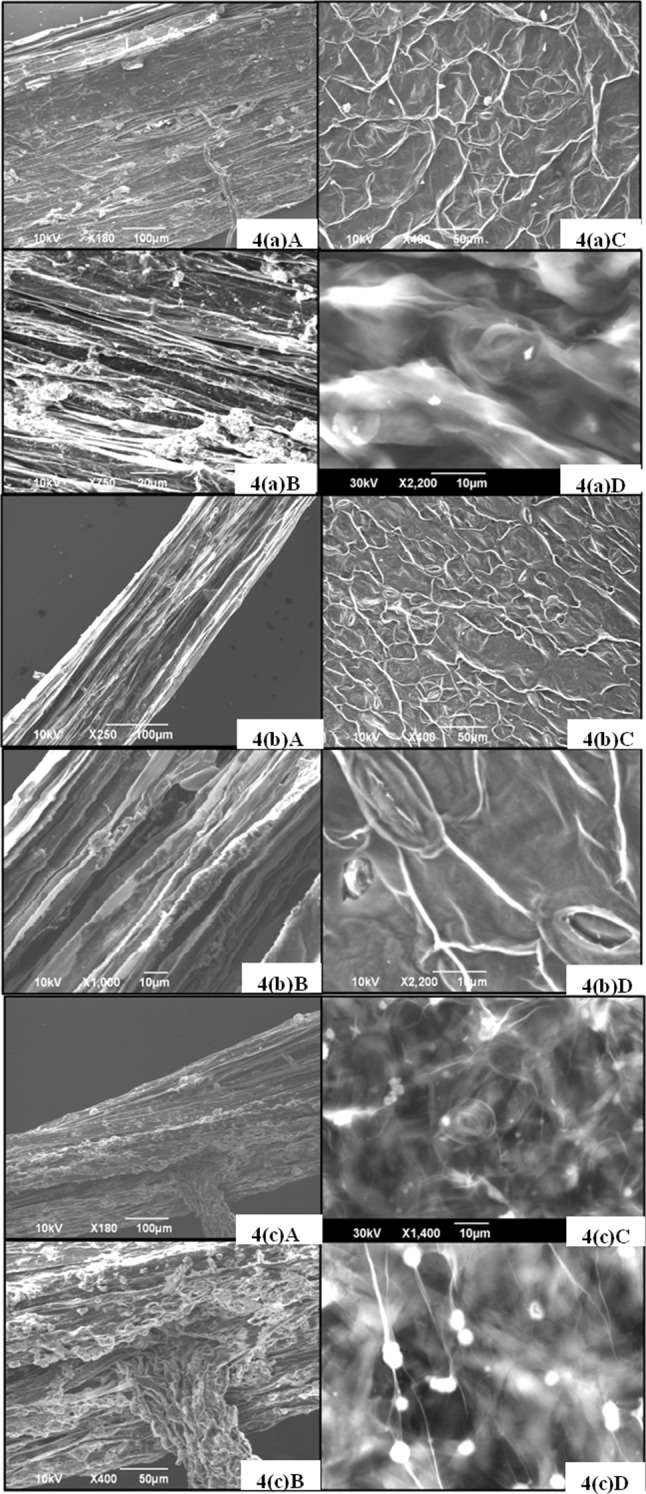
**(a)A** Root Surface of 1500 mg/L CuO treated plant (*scale bar* 100 µm) **(a)B** Root surface of 1500 mg/L treated plant **(a)C** Leaf surface of CuO 1500 mg/L treated plant (*scale bar* 50 µm), **(a)D** Leaf surface of 1500 mg/L treated plant (*scale* 10 µm). **(b)A** Root of *B. juncea* control plant, **(b)B** Root, **(b)C** Leaf, **(b)D** Leaf (*scale* 10 µm). **(c)A** Root Surface of 1500 mg/L TiO_2_ treated plant, **(c)B** Root surface of 1500 mg/L treated plant, **(c)C** Leaf surface of 1500 mg/L TiO_2_ treated plant, **(c)D** Leaf surface of 1500 mg/L treated plant

Anatomy of plants exaggerated with nanoparticles has been studied previously by different microscopic techniques, such as a light microscope, SEM and TEM (Gonzalez-Melendi et al. 2008). In this work, for confirmation of uptake of NPs, SEM analysis of root and leaf samples was performed. A conspicuous aggregation of CuO NPs on root surface of 1500 mg/L treated plants was found [Fig. 4(a)A, B]. The appearance of shining dots in CuO NPs treated plant leafs as shown in Fig. 4(a)C, D was observed. But when root and leaf tissues of control plant was observed no such inconsistency was found in root tissues as indicated in [Fig. 4(b)A, B] also not in the case of control plant leaf as in Fig. 4(b)C, D. But, TiO_2_ NPs treated plant root and leaf as shown in Fig. 4(c)A, B, C, and D, respectively, again the presence of nanoparticle was observed. Therefore, it is hypothesized that NPs are first adhered to root surfaces and through different tissues during transport of water and minerals finally reaches to leaves previously proven by Mueller and Nowack (2008). However, Zhu et al. reported that pumpkin plant (*Cucurbita maxima*); grown in an aqueous medium containing Fe_3_O_4_-NPs, can absorb, translocate and accumulate the particles within the plant tissues (Zhu et al. 2008).

### Effect of NPs on Proline content and lipid peroxidation

Proline has been reported to improve plant’s resistance to oxidative stress by scavenging reactive oxygen species (ROS), by increasing the activity of antioxidative enzymes, therefore, maintaining redox homeostasis (Matysik et al. 2002). Proline content was found to be higher in NP treated plants compared to control as shown in Table 2. A sharp rise in content of proline in the treated plants was recorded with the highest content in roots (1500 mg/L). CuO and TiO_2_ treated roots had 65 and 66% higher proline content than the control. Differential response in MDA content to CuO and TiO_2_ NPs are summarized in Table 2. Amplified accumulation of lipid peroxide is indicative of enhanced production of ROS. The level of MDA content increased in CuO treated roots at 1000 and 1500 mg/L with a significant difference at all concentrations except at 200 mg/L. While, in TiO_2_ treated plants the content was found to be almost 50% less in shoots and 9% less in the roots at 1500 mg/L as compared to control. Although, silver nanoparticle (0, 25, 50, 100, 200 and 400 ppm) had shown a considerable effect on MDA content which was found to be declined in treated seedlings compared to control in 7-day old *B. juncea* seedlings (Sharma et al. 2012). Similarly, Nekrasova et al. observed that in *Elodea densa* plants treated with copper ions and Cu NPs had an enhanced lipid peroxidation up to 120 and 180% of the control; respectively (Nekrasova et al. 2011). Present results are also in accordance with those observed in wheat (14-day old) there were a higher MDA values (4.45 ± 0.4) in CuO treated plant then and control plants (Dimkpa et al. 2012).

### Interrelationship of hydrogen peroxide content and antioxidant enzymes

**Fig. 5:**
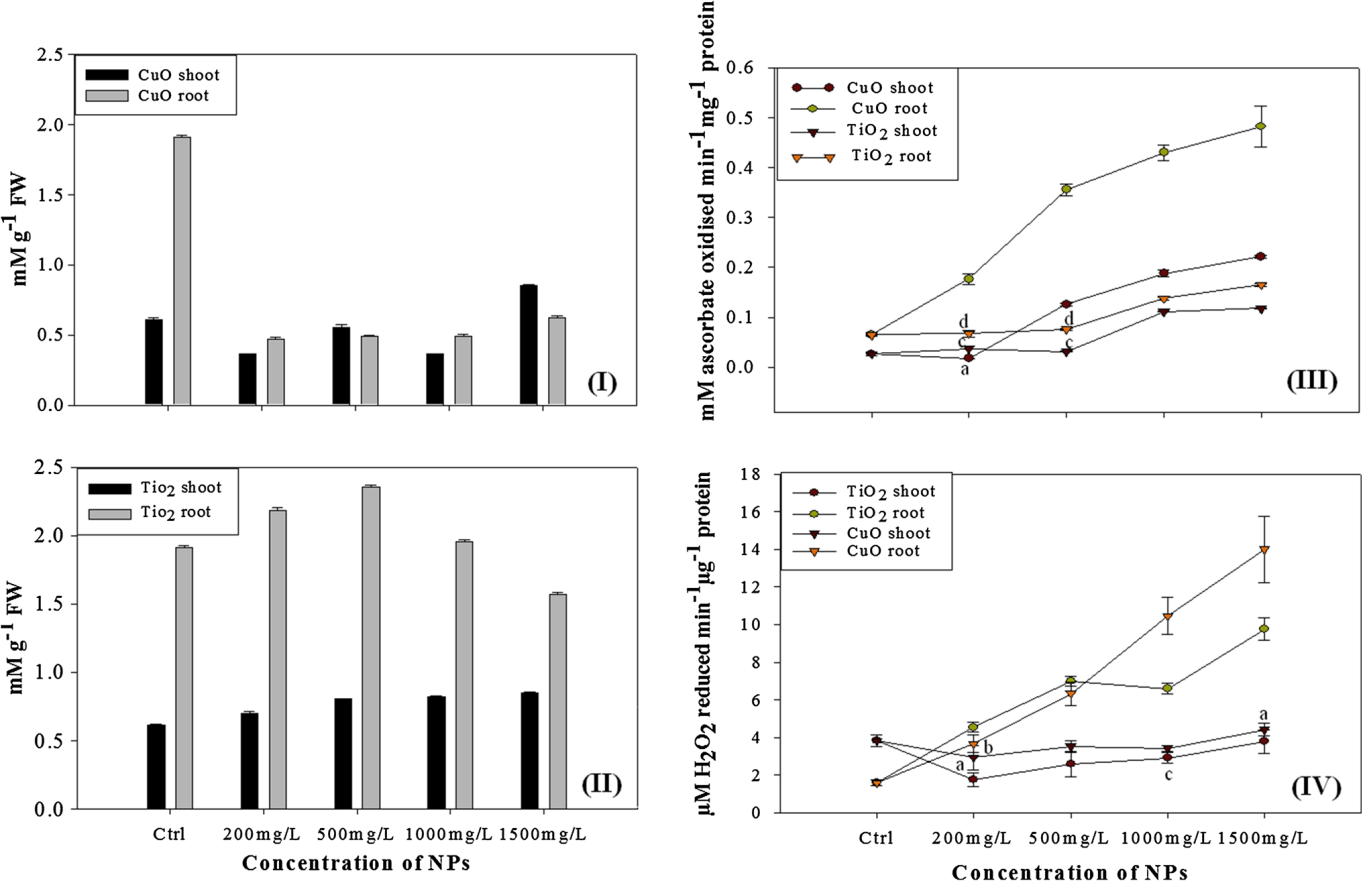
Effect of **I** CuO NPs and **II** TiO_2_ NPs on hydrogen peroxide (H_2_O_2_) content in *B. juncea*. **III** Dynamics of Ascorbate peroxidase (APX) enzyme activities in *B. juncea* exposed to CuO and TiO_2_ NPs. **IV** Dynamics of catalase (CAT) enzyme activities in *B. juncea* exposed to CuO and TiO_2_ NPs after 96 h of treatment. *Bars* indicate means and SD (*n* = 3), *different letters on bars* indicate no significant differences between treated and control plant set in the respective enzyme activity (*p* < 0.001)

Growing evidence suggests that H_2_O_2_ plays a versatile role in plant defense and physiological reaction. It functions as an important signal molecule during plant growth and development. H_2_O_2_ accumulation is maintained at the very low level because of the existence of an antioxidant system in the plant, for elimination of excess H_2_O_2_. It is a major ROS in plants and alteration in the content could be taken as a precise indicator of oxidative stress in *B. juncea*. H_2_O_2_ content in the *B. juncea* shoot was enhanced by both of CuO and TiO_2_ NPs concentrations, leading to significant increase by 28% at 1500 mg/L CuO as shown in Fig. 5(I) while, content was only 19% higher in TiO_2_ treated roots at the same concentration [Fig. 5(II)]. In root tissues of CuO treated plants the content was lower comparable to that in control.

APX is an important antioxidant enzyme system involved in the ascorbate–glutathione cycle occurring in chloroplasts, cytoplasm, mitochondria and peroxisomes (del Rio et al. 2006). In chloroplasts, APX removes H_2_O_2_ using ascorbate as an electron donor. The remarkable changes in the enzymatic activity of APX in shoot and roots of control and nanoparticle treated plantlets have been summarized in Fig. 5(III). The results pertaining to the effect of different concentrations of CuO and TiO_2_ on enzyme activity which was found to be highest in the roots of plants treated with CuO NPs at 1500 mg/L. None of the significant alteration in enzyme activity was observed in the roots of seedlings exposed to TiO_2_ (500 mg/L) but, subsequently at 1000 mg/L and 1500 mg/L the activities increased up to 52 and 60%. In plants (shoots) treated with both of the NPs, had no significant difference up to 200 mg/L but later, increase in the specific enzyme activity was observed, with the highest activity in shoots treated by CuO NPs (1500 mg/L). Similar studies in past have also demonstrated that ionic form of Cu can affect the ascorbate–glutathione pathway in the primary leaves of plants (Cuyers et al. 2000; Palma et al. 2002; Wang et al. 2004). Catalase is another key enzyme in the scavenging of H_2_O_2_ into the water and molecular oxygen. As a result of CuO stress, in roots, CAT activity was found to be higher as compared to control as well as TiO_2_ NPs treated plants [Fig. 5(IV)]. A linear rise in enzyme activity was observed up to 1500 mg/L with a significant difference at all concentrations. A noticeable increase in enzyme activity in roots was observed at 1000 mg/L and 1500 mg/L, i.e., 84 and 88% more than the control. Similarly, in roots of TiO_2_ NPs treated plants the activity was 83% higher than the control. While in the shoot part of the CuO NPs treated plants there was no significant increase in enzyme activity up to 1000 mg/L. In case of TiO_2_ NPs at the same concentration in shoot, the activity was found only 2.5% higher than control. Therefore, it is evident from above results that CuO NPs are responsible for the higher CAT enzyme activity in *B. juncea.* Decline in enzyme is regarded as a general response to many stresses and it is supposedly due to inhibition of enzyme synthesis or a change in the assembly of enzyme subunits (MacRae and Ferguson 1985).

**Fig. 6:**
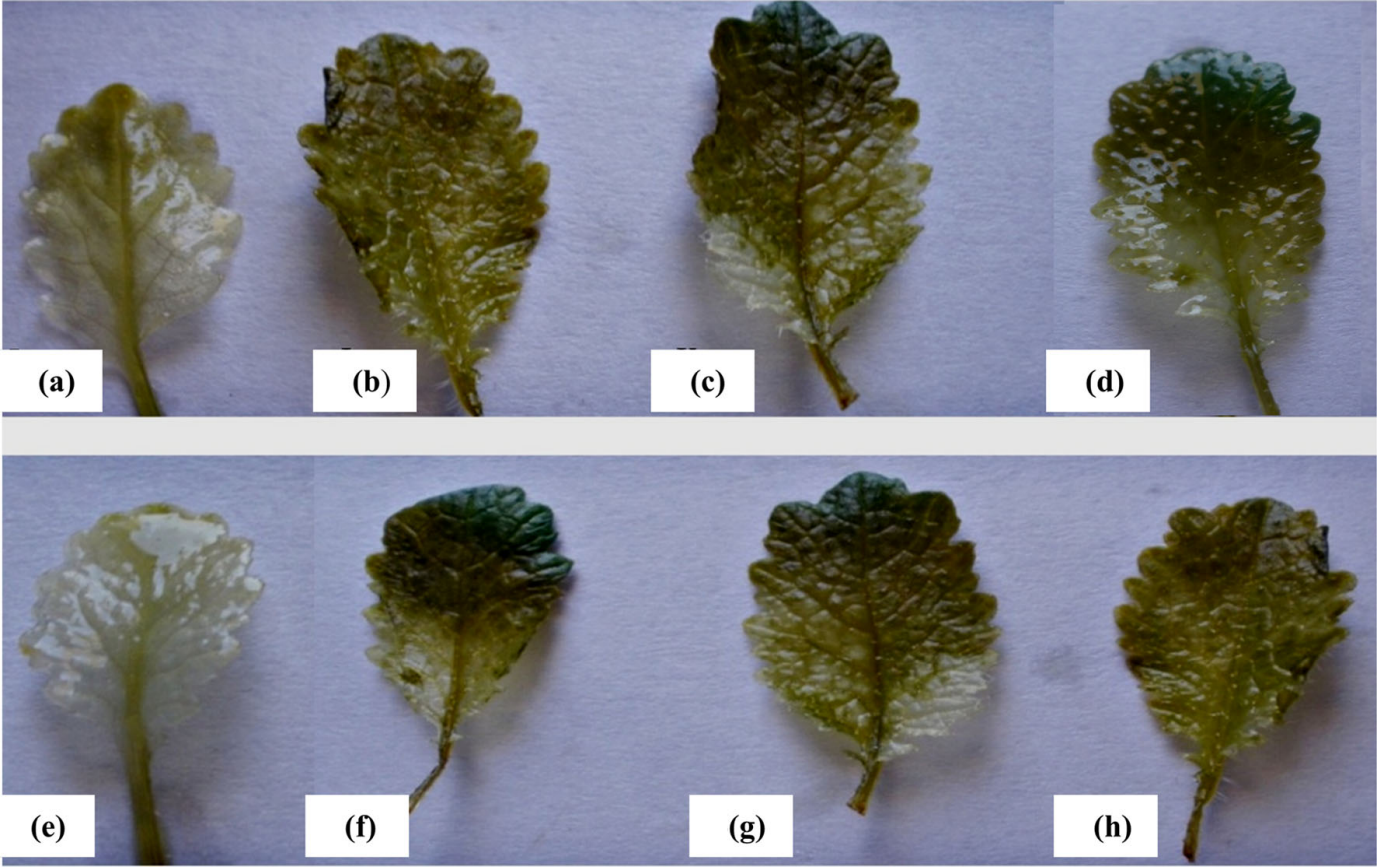
Effect of NPs on accumulation of H_2_O_2_ in leaves tested by means of the ROS sensitive dye DAB in *B. juncea* seedlings. **a**, **e** Control leaves showing no polymerized product as almost transparent visibility. **b–d** CuO treated plant leaves (200, 1000, 1500 mg/L) with *greenish brown spots* as compared to control plant leaves and **f–h** TiO_2_ treated plant leaves showing spots (200, 1000, 1500 mg/L), respectively

The excessive presence of H_2_O_2_ was induced by CuO and TiO_2_ NPs, detected by means of the ROS-sensitive dye 3, 3′-diaminobenzidine (DAB) that polymerizes in the presence of ROS species. The location of insoluble deep radish brown polymerization compound produced when DAB reacts with H_2_O_2_ could be visualized by naked eyes. Imaging of deep greenish brown polymerization product as an indication of accumulation of H_2_O_2_ in *B. juncea* was observed. In control plant leaf (Fig. 6a, e) no distinctive deep radish brown polymerization of H_2_O_2_ was observed. The plants exposed to 200 mg/L CuO (Fig. 6b), and TiO_2_ (f) at the same concentration had not shown any significant amount of polymerization. But as shown in Fig. 6d, 6 h, higher levels of CuO and TiO_2_ (1500 mg/L) triggered production of more ROS. The excess of ROS formation at 1500 mg/L may be indicative of concentration-dependent ROS generation.

SOD enzyme activity had the similar pattern as shown by the APX and CAT. Its level significantly increased in the roots of CuO NPs augmented plants (87% higher than the control at 1500 mg/L NP) but no significant increase was observed in the roots and shoot of TiO_2_ treated plants (Figure Supplement-S2).

In CuO NPs treated *B. juncea* (at 500 mg/L) the activity was abruptly found to be 80% higher than the control and after which there was a decline in activity. Kim et al. observed that CuO and ZnO NPs were more actively accumulated in root tissues of *Cannabis sativa*, while antioxidant enzyme (CAT, SOD and peroxidase) activities in plants treated with these nanoparticles increased by a factor of 1.5–2.0 (Kim et al. 2012). It was observed that by treatment of plant by both NPs increased the Cu and Zn concentrations in root tissues and formed agglomerates, either with themselves or with other cellular materials within the cells. Similarly, in another study, plants grown with ZnO NPs had reduced CAT activity (Dimkpa et al. 2012). While silver nanoparticle treatment to 7-day old *B. juncea* had been found to induce the activities of antioxidant enzymes (APX, peroxidase and CAT), resulting to reduced reactive oxygen species level (Sharma et al. 2012). Our findings were in line with these studies, thereby confirming the accumulation of NPs in plant systems, which disrupt the metabolism by inducing ROS generation and subsequent alteration of oxidative stress enzymes in plants. Based on findings, we suggest that levels of antioxidant enzymes is a predictive biomarker for oxidative stress in *B. juncea* exposed to CuO and TiO_2_ NPs. Amongst the stress enzymes, APX has a much higher affinity to H_2_O_2_ than CAT suggesting that they have different roles in the scavenging of ROS. In this study, the CAT activity was found decreasing as the concentration of the CuO increased. The level of H_2_O_2_ content was also increased in induced stress condition in treated samples due to the lower activity of CAT. APX being responsible for maintaining the low levels of H_2_O_2_ while CAT is responsible for the removal of its excess.

In conclusion, *B. juncea* displayed a negative effect of high nanoparticle concentration on plant growth through generation of oxidative stress. CuO NPs are known to have phytotoxic effects in plant cells because of aggregation, causing cell death and accumulated ROS in a dose-dependent manner. On the other hand, TiO_2_ NPs showing a supportive role in growth could be incorporated to fertilizers for the crop improvement program. Inhibition of plant growth by CuO NPs may be due to physical interaction between nanoparticles and plant cellular transport mechanism via blockage of the intercellular spaces in the cell wall or between cells through plasmodesmata. However, it could accumulate lower concentration of nanoparticles under low levels of stress and exhibited significant tolerance against stress, possibly due to efficient and well-coordinated defense mechanisms at the root and the shoot level due to modulation of antioxidative enzymes.

## Electronic supplementary material

Below is the link to the electronic supplementary material.

Supplementary material 1 (DOCX 605 kb)
