# Supplementary material for: Phytotoxicity and oxidative stress perspective of two selected nanoparticles in Brassica juncea
Source: 3 Biotech. 2016 Nov 15;6(2):244. doi: 10.1007/s13205-016-0550-3 (PMC5110483; doi:10.1007/s13205-016-0550-3)
Supplement: Supplementary file 1 — Supplementary material 1 (DOCX 605 kb) [file 13205_2016_550_MOESM1_ESM.docx]

**Supplementary information**


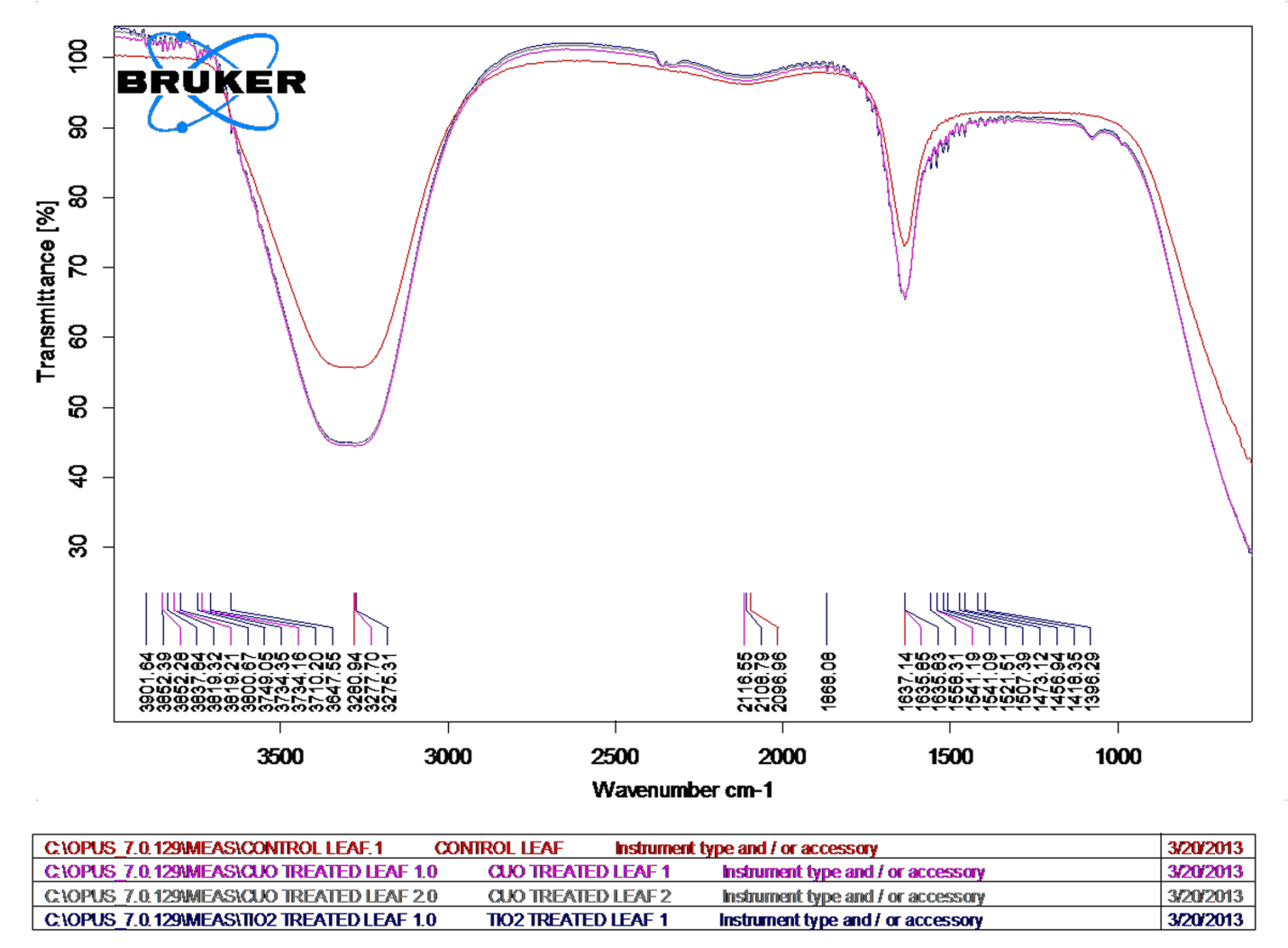


**FigureS1:**FTIR spectrum of plant leaf samples: *B. juncea* exposed to 5 different concentrations of CuO and TiO_2_ NPs after 96 h of treatment.

**
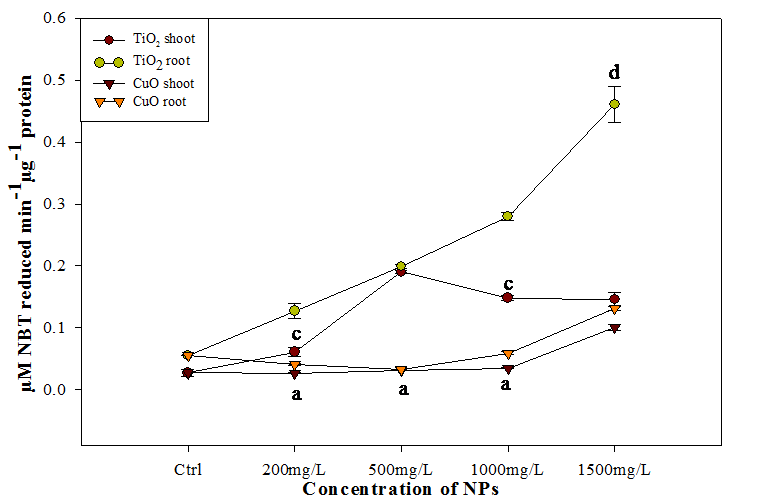
**

**FigureS2:** Superoxide dismutase (SOD) enzyme activities: 5 different concentrations of CuO and TiO_2_ NPs after 96 h of treatment.
